# Determinants of Equity in Coverage of Measles-Containing Vaccines in Wales, UK, during the Elimination Era

**DOI:** 10.3390/vaccines11030680

**Published:** 2023-03-17

**Authors:** Malorie Perry, Simon Cottrell, Michael B. Gravenor, Lucy Griffiths

**Affiliations:** 1 Vaccine Preventable Disease Programme and Communicable Disease Surveillance Centre, Public Health Wales, 2 Capital Quarter, Tyndall Street, Cardiff CF10 4BZ, UK; 2 Population Data Science, Health Data Research UK, Swansea University Medical School, Swansea SA2 8PP, UK

**Keywords:** vaccination, immunisation, socioeconomic factors, measles, MMR, measles, mumps and rubella vaccine

## Abstract

In the context of the WHO’s measles and rubella elimination targets and European Immunization Agenda 2030, this large cross-sectional study aimed to identify inequalities in measles vaccination coverage in Wales, UK. The vaccination status of individuals aged 2 to 25 years of age, alive and resident in Wales as of 31 August 2021, was ascertained through linkage of the National Community Child Health Database and primary care data. A series of predictor variables were derived from five national datasets and all analysis was carried out in the Secure Anonymised Information Linkage Databank at Swansea University. In these 648,895 individuals, coverage of the first dose of measles-containing vaccine (due at 12–13 months of age) was 97.1%, and coverage of the second dose (due at 3 years and 4 months) in 4 to 25-year-olds was 93.8%. In multivariable analysis, excluding 0.7% with known refusal, the strongest association with being unvaccinated was birth order (families with six or more children) and being born outside of the UK. Living in a deprived area, being eligible for free school meals, a lower level of maternal education, and having a recorded language other than English or Welsh were also associated with lower coverage. Some of these factors may also be associated with refusal. This knowledge can be used to target future interventions and prioritise areas for catch up in a time of limited resource.

## 1. Introduction

The 2012 WHO Global Measles and Rubella Strategic Plan outlined the aim to achieve elimination of measles and rubella in at least five of the six WHO regions by the end of 2020 [1]. In 2018, the UK lost its measles elimination status, and since 2020 endemic transmission remains re-established [2]. Although there has not been a confirmed case of rubella in Wales (one of the four nations of the UK) since 2010, there have been regular and sometimes large outbreaks of measles [3,4,5]. A milestone achievement in the 2012 strategy was to ensure countries have at least a 95% uptake of two routine doses of measles- and rubella-containing vaccine by 2020. In line with these aims, the Wales Measles Elimination Task Group Action Plan 2019–2021 specifically highlighted the importance of increasing measles, mumps and rubella (MMR) vaccination coverage in young people [6]. 

Public Health Wales has produced COVER (Coverage of Vaccination Evaluated Rapidly) reports for over 30 years [7]. These reports present uptake of all routine childhood immunisations up to 16 years of age. These figures are fed back to vaccination providers to guide service improvements and requirements for catch-up. Data for these reports come from the National Community Child Health Database (NCCHD), which is a population register of all children in Wales registered with the National Health Service (NHS). Primary care doctors and nurses, school nurses and immunisation teams administering vaccines send completed vaccination forms to their health board child health office, detailing vaccinations that have been given. This information is then entered into the health board child health database, with the records extracted on a monthly basis and combined to form the NCCHD. The first dose of MMR is due at 12–13 months of age with a second routine dose at 3 years and 4 months, before school entry. Vaccination status checks are encouraged at routine primary care appointments, on entry to primary and secondary school, and alongside administration of teenage immunisations. 

Routine reporting in Wales has shown that national coverage of one dose of MMR reported at two years of age has ranged between 86% and 98% over the last 20 years, whilst coverage of two doses at five years of age has varied between 71% and 94% [7]. Currently coverage is generally high; however, coverage in teenagers is lower and varies by region. Coverage in those aged older than 16 years is not routinely reported due to archiving of NCCHD data around this age. At an ecological level, there is lower vaccine uptake in more deprived areas compared to less deprived areas across all age groups [8]. Equitable access and coverage of vaccinations has been highlighted in the WHO European Immunization Agenda 2030 [9]. Socioeconomic factors are often associated with vaccination coverage for routine childhood immunisations. In developed countries, areas experiencing poverty, families that have a lower income, parents with a lower level of education and those experiencing unemployment are generally associated with lower vaccine uptake [10,11]. In contrast, higher education status [12] and higher income [13] have also been shown to be associated with lower uptake of vaccines in some populations. The association between poverty and low vaccine coverage is also seen in many low and middle income countries [14]. Vaccination coverage also appears to be lower in children resident in large or single parent households [11,15,16]. Demographic factors such as ethnicity, age of mother, country of birth, religion and gender have also been shown to be predictors of vaccination status [14,17,18,19,20]. 

Large ecological studies looking at routine childhood immunisations are still rare, and specific reasons for low uptake in Wales have not been previously explored. In this study we used data linkage of national datasets to identify factors associated with lower coverage of measles-containing vaccine, with the aim that this knowledge can be used to investigate what the barriers are for uptake of vaccination, develop interventions and prioritise areas for catch-up in a time of limited resources. 

## 2. Materials and Methods

Analyses were completed within the Secure Anonymised Information Linkage (SAIL) Databank held at Swansea University [21]. A cohort of individuals aged 2 to 25 years of age, alive and resident in Wales as of 31 August 2021, was created using the Welsh Demographic Service Dataset. Individuals who do not have a record in the NCCHD or were registered to a GP that does not submit data to SAIL were excluded. Approximately 80% of GP practices in Wales submit data to SAIL [22]. 

Measles vaccination status was assigned using an extract from the NCCHD, supplemented by Read coded vaccination status data from primary care GPs. MMR, measles and rubella (MR) and single antigen measles vaccination were all considered valid vaccinations in this analysis. In line with UK guidance, the first dose of vaccine had to have been given at 12 months of age or later with the second dose given at least one month after the first at 15 months of age or later [23]. 

A series of independent variables were identified to test for association with vaccination coverage. Gender, age as of 31 August 2021, month of birth, mothers’ age at birth, health board of residence, and age first moved to Wales were taken from the Welsh Demographic Service Dataset. Urban/rural classification and deprivation quintile of residence were derived as described previously [24]. Broad ethnic group was derived from the Office for National Statistics 2011 census, with information taken from the Education Wales Schools and Pupils Dataset or primary care GP record, if census data were unavailable. Total number of primary care visits in the 1 September 2020 to 31 August 2021 year and age first registered with a primary care GP in Wales were calculated using data from primary care GPs, and flags were derived for learning disability, diagnosed sight loss and hearing loss based on published primary care Read code sets [25,26]. Mothers’ unique identifier, birth order, maternal smoker flag and premature status (born before 37 weeks’ gestation) were taken from the NCCHD. A flag for ever being eligible for free school meals, attendance at a special school or ever being excluded from school was taken from the Education Wales Schools and Pupils Dataset. Information on mothers’ highest qualification was taken from the 2011 census. Religion was as recorded in census data, otherwise as recorded in data from primary care GPs. Where there were contradicting values, the most recent record was kept. Mothers’ religion was used as default; otherwise, where this was missing, the child’s recorded religion was used. Country of birth (COB) was derived from the Office for National Statistics Annual District Birth Extract. If a child was born in Wales they appear in this data; otherwise, this information was taken from the 2011 census or data from primary care GPs. Where a child’s COB was unknown, mother’s COB was used. Mother and child’s recorded language was taken from census data, and where this was unavailable, language data from primary care GPs was used. If this information was not recorded in either dataset but they were born in Wales it was assumed English/Welsh was a primary language. A Charlson Comorbidity Index score was created using data from primary care GPs based on published Read code sets [27]. Previous vaccinations (three doses of pertussis-containing vaccine, one of pneumococcal vaccine and two of rotavirus vaccine) as outlined in the UK schedule were derived using the same methods used for measles-containing vaccine.

The odds of being vaccinated with one and two doses of measles-containing vaccine were calculated, with independent variables considered significant at the 0.05 level. In a multivariable analysis of those aged 4 to 25 years, records with missing information were dropped. The maternal smoker flag was dropped due to a high proportion of missing data. Mothers’ recorded language and age first moved to Wales were excluded from the multivariable model due to co-linearity with child’s recorded language and age first registered with a Wales GP, respectively. The final model was constructed stepwise in order of strength of association as indicated by the univariable analysis; variables which did not improve the Akaike Information Criterion score were dropped. 

Unvaccinated individuals with a vaccine refusal Read code (68NY., 68NB., 68NP., 68NR., 68Nb., 68Na., 8I3x., 68N6., 68NM.) on their GP record were excluded from the equality analysis and described separately. 

## 3. Results

### 3.1. Coverage of Measles-Containing Vaccine in the Study Population

**Figure 1:**
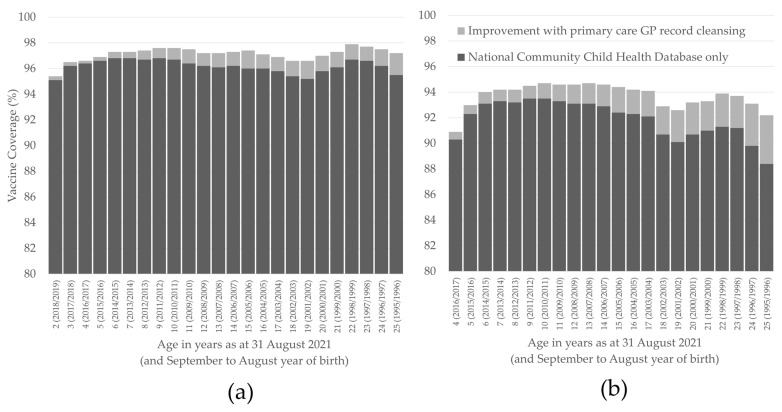
Coverage of one (**a**) and two (**b**) doses of measles-containing vaccine in those aged 2 to 25 years of age, alive and resident in Wales as of 31 August 2021. The improvement in coverage from reconciling the National Community Child Health Database and primary care GP data is also shown.

There were 795,734 individuals aged 2 to 25 years of age, alive and resident in Wales as of 31 August 2021. Of these, 35,254 did not have a record in the NCCHD and a further 111,585 were not registered with a GP who submits data to the SAIL Databank. Using NCCHD data only, coverage of one dose of measles-containing vaccine in these 648,895 remaining individuals was 96.2% and coverage of two doses in those aged 4 to 25 years of age was 92.0%. After reconciling with GP data, coverage increased to 97.1% for one dose and 93.8% for two doses (Figure 1). Of those who were vaccinated, 1620 had received measles-containing vaccines other than MMR for their first dose and 2781 had received measles-containing vaccines other than MMR for their second dose. The majority of non-MMR measles vaccines were given to those aged 15 to 21 years (with the highest proportion received by 20-year-olds, 1.3%). The proportion of all measles vaccines received that were non-MMR was under 0.5% in all other age groups. 

### 3.2. Determinants of Measles Vaccination Coverage

After exclusion of 4688 individuals with vaccine refusal codes, there were 644,207 individuals aged 2 to 25 years in the equity study population. In a univariable analyses, month of birth was the only variable that was not significantly associated with vaccination uptake of either dose. Having had previous vaccinations was strongly associated with having had at least one dose of measles-containing vaccine; OR 177.45 (95% CI 162.99–193.60) for pneumococcal vaccine, OR 100.25 (95% CI 96.52–104.14) for three doses of pertussis vaccine and OR 27.60 (95% CI 25.82–29.50) for two doses of rotavirus vaccine. 

Age first registered with a primary care GP in Wales was most strongly associated with vaccine coverage, with those first registering at secondary school age (12 to 16 years of age) least likely to be recorded as vaccinated, compared to those born in Wales. Those born outside of the UK were less likely to be vaccinated, OR 0.07 (95% CI 0.06–0.07) for one dose. For groups with at least 100 persons, coverage of one dose was under 80% in those born in Romania, Bulgaria, Syria, Lithuania, Turkey, Slovakia, Czech Republic, Nigeria, Zimbabwe, Iraq, South Africa and Asia (not otherwise specified). Coverage was also higher in those who had English or Welsh recorded as a language, OR 8.45 (95% CI 7.88–9.06) for one dose. For groups with at least 100 persons, coverage of one dose was under 80% in those recorded as speaking Bulgarian, Romanian, Lithuanian, Russian, Hungarian, Slovak, Italian and Spanish. There was also association with ethnicity and coverage, with those who were in a combined Black, Asian, Mixed or other ethnic group having lower coverage than those in the combined White ethnic group. In the univariable analysis those with a recorded religion of Buddhism, Islam, Pagan or other religions were less likely to be vaccinated than those who stated they had no religion. 

Females were more likely to be vaccinated than males, OR 1.09 (95% CI 1.05–1.12) for one dose. Mothers who were older (36 years and over) and younger (under 17) when giving birth were less likely to have children who were vaccinated, as well as those born in to families with more children (OR 0.16 95% CI 0.14–0.18 if sixth or greater compared to first born). There was variation by health board and deprivation quintile of residence, with vaccination less likely in more deprived areas. Coverage was also lower in urban areas, compared to rural areas, OR 0.74 (95% CI 0.71–0.77) for one dose. Those who have ever been eligible for free school meals were less likely to be vaccinated, OR 0.86 (95% CI 0.82–0.89) as well as those who were born to mothers who smoked during pregnancy (OR 0.76 95% CI 0.69–0.84) and mothers who had no qualifications compared to those with at least GCSE qualifications. People were less likely to be vaccinated with two doses if they had a school exclusion record (OR 0.76 95% CI 0.72–0.80), although this association was not seen with one dose. This association was stronger for those with a permanent exclusion record compared to a temporary exclusion record.

There was no association with vaccination and premature birth for dose one but coverage of two doses was significantly lower, OR 0.92 (95% CI 0.87–0.97). Those who consulted with their GP at least once between 1 September 2020 and 31 August 2021 were more likely to be vaccinated, and coverage was significantly higher in those with recorded comorbidities. Those with chronic pulmonary disease, renal disease and uncomplicated diabetes were significantly more likely to be vaccinated, and those with liver disease, peptic ulcer and rheumatic disease were significantly less likely to be vaccinated. Coverage of at least one dose in those with hearing loss or sight loss was higher than the rest of the study population; coverage of two doses in those with sight loss was not significantly different. Coverage of two doses in those with a learning disability was lower than the general population, OR 0.62 (95% CI 0.55–0.71), although there was no difference for one dose. Those who attend, or have attended, a special school had lower coverage of one and two doses. 

The univariable analyses including the full cohort can be found in Supplementary Table S1.

The variables included in the final model are presented in Table 1. The cohort was restricted to those aged 4 to 25 years without missing information across all variables, producing a study population of 419,405. This restricted cohort had higher vaccine coverage overall, and led to some notably different estimates in the univariable analyses. Although having a comorbidity score of one was associated with higher vaccination coverage compared to those with no comorbidities, those with a score of three or more were less likely to be vaccinated in the restricted cohort. Also, younger mothers (aged under 17) in the restricted cohort were more likely to have vaccinated children compared to the univariable analysis in the full cohort, which showed they were less likely. 

In the multivariable analysis, after controlling for other factors, the strongest association with vaccination uptake was birth order (OR 0.21 95% CI 0.17–0.26 for one dose if sixth or greater compared to first born) and being born outside of the UK (OR 0.21 95% CI 0.18–0.25 for one dose). Living in a more deprived area of residence was still associated with lower coverage but the association was not as strong. The association with recorded language, free school meal eligibility and mothers’ highest qualification was also slightly reduced. Having a comorbidity score of 3 or more was no longer significant. However, the biggest difference was seen in ethnicity, where those in the combined Asian ethnic group were more likely to be vaccinated with at least one and two doses after controlling for other factors (OR 1.58 (95% CI 1.27–1.97) for at least one dose), and those in the combined Black ethnic group were no longer significantly less likely to be vaccinated. Differences were also seen in those who had recorded religion of Islam, who were more likely to be vaccinated with one and two doses after controlling for other factors (OR 1.45 (95% CI 1.13–1.87) for at least one dose). 

### 3.3. Individuals Refusing Measles Vaccination

A total of 4688 individuals aged 2 to 25 years had a vaccine refusal code on their GP record (0.7% of the full study population). Of these, 1814 had received one dose of measles-containing vaccine but not two. The proportion of recorded refusals were highest in those aged 2 and 3 years (over 1.0%). 

In a univariable analysis comparing individuals who had received one dose of measles-containing vaccine, with those who had a vaccine refusal code; those resident in urban areas were more likely to be vaccinated than have a refusal code compared to those resident in rural areas (OR 1.51 95% CI 1.40–1.63). Additionally, those with a recorded language of English or Welsh were more likely to be vaccinated (OR 1.53 95% CI 1.27–1.81), as well as those in less deprived areas compared to more deprived areas (OR 1.19 95% CI 1.06–1.35). Children born to mothers who were over 30 years of age were less likely to be vaccinated than have a refusal code, compared to those aged 26–30 years (OR 0.54 95% CI 0.46–0.62 for mothers aged over 40), as well as those with more siblings (OR 0.31 95% CI 0.24–0.40 if sixth or greater compared to first born) and those eligible for free school meals (OR 0.83 95% CI 0.76–0.91). Having a recorded religion of Buddhism (OR 0.27 95% CI 0.17–0.44) or Paganism (OR suppressed due to small numbers) was associated with being less likely to be vaccinated than have a refusal code, compared to those with no religion, whereas having a recorded religion of Christian was associated with being more likely to be vaccinated (OR 1.22 95% CI 1.11–1.34). Those in the combined Asian ethnic group were more likely to be vaccinated compared to those in the combined White ethnic group (OR 2.21 95% CI 1.57–3.24), whereas those in the combined Black ethnic group (OR 0.65 95% CI 0.45–0.98) or combined Mixed ethnic group (OR 0.70 95% CI 0.57–0.87) were less likely to be vaccinated than have a refusal code.

## 4. Discussion

Measles vaccination uptake in this cohort of children and young adults in Wales is reassuringly high, with coverage of one dose over 95% in all NHS-registered children aged 2 to 25 years. However, potential remains for outbreaks of measles where unvaccinated individuals are clustered. All routine childhood vaccinations in Wales should be recorded in the child health system until 16 years of age to manage appointment call and re-call and enable accurate reporting. However, we have seen that administrative records are not always correct, and reconciling multiple data systems may help improve accuracy. Despite high coverage, minor improvements in some age groups may be the difference between reaching the 95% coverage target or not. Although the oldest individuals in this analysis would have been scheduled for vaccination in the latter years of the decline in MMR uptake seen following the Wakefield scandal, coverage appears high. However, the young adults who were young children at the time of the negative publicity might explain some of the significantly reduced odds of vaccination in those aged 19 years and older. This analysis may also exclude a number of individuals who are not registered with the NHS, and therefore not included in the datasets used to produce these figures.

MMR coverage is frequently reported as a measure of the proportion of the population protected from measles infection. These analyses have identified that over 4000 single (or duel MR) antigen measles doses had been received by those in the study cohort. However, the receipt of non-MMR vaccines has decreased in younger age groups. Some of these records may be miscoded and validation of the type of vaccination received would be necessary to have accurate records of which viruses individuals are protected against.

A small proportion of the study population (0.7%) had a GP Read code indicating measles vaccine refusal. A higher proportion of those in younger age groups had a refusal code, which could be an indication of a recent increase in vaccine hesitancy. However, this trend could also be due to improvement in coding over time. Although this study tries to focus on factors associated with low coverage in those who have not actively refused vaccination, there is suggestion of variation in refusal by different characteristics, some of which, such as residing in rural areas, appear to be associated with refusal but not other reasons for being unvaccinated. Monitoring refusals would be beneficial to highlight any concerns or mistrust as early as possible [28]. The USA has seen a recent increase in exemptions for MMR vaccine due to religious, philosophical or personal reasons, which may be contributing to a resurgence in cases [29].

Excluding those with known refusal, we have seen that inequitable coverage is particularly prevalent in households with more children and for those born outside of the UK. Living in a deprived area, being eligible for free school meals, lower level of maternal education, and having a recorded language other than English or Welsh were also associated with lower coverage. These factors are similar to those mentioned in previously published literature [10,12,15,30]. Lower coverage persists in deprived urban areas, and factors relating to deprivation are complex and hard to separate out. 

Evidence from this study is useful to develop tailored interventions; for example, community health care visits [31], which in this case could be prioritised for large households with multiple unvaccinated children, or joint scheduling for siblings that require catch-up. Having had previous vaccines meant there was a higher chance of having had measles-containing vaccine, suggesting it may be efficient for catch-up campaigns to target more than one vaccine programme. Improving accessibility of resources and using tailored public health messaging may reduce inequities [32]. In addition, using the WHO Tailoring Immunisation Programmes approach can help us understand specific barriers in communities identified as having lower coverage [33,34]. 

A recent review has suggested migrants are half as likely to be vaccinated compared to non-migrants [35]. Challenges specifically relating to migrants who have transited through a number of countries, and refugees, include lack of information on vaccination status at arrival, fear of registration with medical authorities and lack of coordination between public health authorities of neighbouring countries [36]. It is likely that recording of immunisations in those who were on vaccination schedules different to the UK is difficult and parents often do not have evidence of their child’s previous vaccinations, which makes entering dates into the system, and scheduling further doses, challenging. UK guidance indicates restarting a vaccine course if vaccination history is uncertain [37]. Tailoring immunisation services to ensure there are no language barriers when carrying out vaccination status checks and ensuring flexible systems for recording immunisations from overseas could be beneficial. Low vaccination coverage in Eastern European communities has been linked to measles outbreaks in the UK, with language, literacy and trust of health care providers identified as potential barriers [38]. Building trusting relationships with minority groups such as Gypsies, Travellers and Roma may also improve utilisation of health care services including uptake of vaccination [39].

There are limitations to this study. Some individuals will not be registered with NHS health services, and those who do not have a NCCHD record were excluded, which will affect those who first resided in Wales after 16 years of age. Additionally, some vaccinations recorded in primary care GP data, but not on the NCCHD record, for older ages may be due to catch-up immunisations given more recently. There is the possibility of ‘ghost records’ for those who have moved away and not notified the system. The multivariable analysis was restricted to those without missing information, which disproportionately affected some groups. This analysis would exclude those families who moved to Wales since 2011 when the census took place, as variables such as mothers’ highest education level were derived from census data only. The higher vaccine coverage and reduction in effects that were seen in the multivariable analysis may therefore be due to this restricted cohort only including those who have been settled in Wales for a longer time period. It is challenging to draw conclusions around those factors, which showed different associations in the univariable analyses when using the full and restricted study population, including comorbidities and mothers’ age. Additionally, some data may not reflect the current status of an individual as it may be out of date. This includes information taken from the 2011 census and information on language, as even if a language other than English or Welsh is recorded, a person could be bilingual or have sufficient understanding of English or Welsh to access services and make an informed decision around vaccination. However, this analysis is still a useful indicator to highlight areas at risk of outbreaks and where coverage could be improved. This is a large population study that has been able to provide new evidence on a number of characteristics associated with measles vaccination coverage in Wales.

Reducing inequalities in vaccination coverage remains key for preventing measles outbreaks and reaching the WHO measles elimination targets [1]. Disruption to routine vaccine schedules during the COVID-19 pandemic may have exacerbated the inequalities reported here, making the need for catch-up activities even more pressing [40]. Reported measles cases in Europe decreased from mid-2020 [41], but now that travel restrictions have been fully lifted, the likelihood of a resurgence in cases is high and identifying/reducing inequalities in vaccine coverage should remain a priority.

## Figures and Tables

**Table 1:** Uptake of one or two doses of measles-containing vaccine in those aged 4 to 25 years alive and resident in Wales as of 31 August 2021, without a vaccine refusal code, by individual characteristics. Odds Ratios and 95% Confidence Intervals are also presented. Analysis restricted to those with complete information across all variables.

Characteristic	Category	One Dose of Measles-Containing Vaccine (4–25 Year Olds)					Two Doses of Measles-Containing Vaccine (4–25 Year Olds)				
		Vaccinated (n)	Population (n)	Uptake (%)	OR (95% CI)	aOR (95% CI)	Vaccinated (n)	Population (n)	Uptake (%)	OR (95% CI)	aOR (95% CI)
Gender	Male	213,518	215,626	99.0	Baseline	Baseline	208,567	215,626	96.7	Baseline	Baseline
	Female	202,068	203,779	99.2	1.17 (1.09–1.24)	1.11 (1.03–1.18)	197,973	203,779	97.2	1.15 (1.11–1.20)	1.11 (1.07–1.15)
Age cohort	Primary school (4–11)	132,916	133,857	99.3	Baseline	Baseline	130,258	133,857	97.3	Baseline	Baseline
	Secondary school (12–16)	109,335	110,028	99.4	1.12 (1.01–1.23)	1.38 (1.24–1.52)	107,809	110,028	98.0	1.34 (1.27–1.42)	1.60 (1.51–1.69)
	College (17–18)	40,779	41,175	99.0	0.73 (0.65–0.82)	0.90 (0.80–1.02)	39,946	41,175	97.0	0.90 (0.84–0.96)	1.08 (1.01–1.16)
	University (19–21)	54,384	55,079	98.7	0.55 (0.50–0.61)	0.70 (0.63–0.78)	52,902	55,079	96.0	0.67 (0.64–0.71)	0.83 (0.78–0.88)
	Young adults (22–25)	78,172	79,266	98.6	0.51 (0.46–0.55)	0.66 (0.60–0.73)	75,625	79,266	95.4	0.57 (0.55–0.60)	0.73 (0.69–0.77)
Health board of residence	HB1	81,805	82,299	99.4	Baseline	Baseline	80,763	82,299	98.1	Baseline	Baseline
	HB2	57,912	58,599	98.8	0.51 (0.45–0.57)	0.51 (0.45–0.57)	56,282	58,599	96.0	0.46 (0.43–0.49)	0.46 (0.43–0.49)
	HB3	43,403	44,002	98.6	0.44 (0.39–0.49)	0.44 (0.39–0.49)	42,355	44,002	96.3	0.49 (0.46–0.52)	0.47 (0.44–0.50)
	HB4	6801	6902	98.5	0.41 (0.33–0.51)	0.38 (0.31–0.48)	6636	6902	96.1	0.47 (0.42–0.54)	0.45 (0.39–0.51)
	HB5	76,485	77,197	99.1	0.65 (0.58–0.73)	0.62 (0.55–0.69)	74,573	77,197	96.6	0.54 (0.51–0.58)	0.54 (0.51–0.58)
	HB6	79,422	80,048	99.2	0.77 (0.68–0.86)	0.76 (0.68–0.86)	77,768	80,048	97.2	0.65 (0.61–0.69)	0.66 (0.62–0.71)
	HB7	69,758	70,358	99.1	0.70 (0.62–0.79)	0.80 (0.71–0.91)	68,163	70,358	96.9	0.59 (0.55–0.63)	0.64 (0.60–0.68)
Deprivation quintile of residence	Most deprived	97,159	98,266	98.9	Baseline	Baseline	94,183	98,266	95.8	Baseline	Baseline
	2	87,377	88,209	99.1	1.20 (1.09–1.31)	1.06 (0.97–1.17)	85,432	88,209	96.9	1.33 (1.27–1.40)	1.15 (1.09–1.21)
	3	75,674	76,343	99.1	1.29 (1.17–1.42)	1.10 (1.00–1.23)	74,185	76,343	97.2	1.49 (1.41–1.57)	1.16 (1.10–1.23)
	4	72,632	73,239	99.2	1.36 (1.23–1.51)	1.11 (0.99–1.23)	71,339	73,239	97.4	1.63 (1.54–1.72)	1.18 (1.11–1.26)
	Least deprived	82,744	83,348	99.3	1.56 (1.41–1.73)	1.13 (1.01–1.26)	81,401	83,348	97.7	1.81 (1.72–1.91)	1.22 (1.15–1.30)
Ethnic group	White	392,908	396,329	99.1	Baseline	Baseline	384,621	396,329	97.0	Baseline	Baseline
	Other	1817	1868	97.3	0.31 (0.24–0.42)	1.08 (0.77–1.54)	1745	1868	**93.4**	0.43 (0.36–0.52)	1.08 (0.86–1.36)
	Asian	9762	9900	98.6	0.62 (0.52–0.73)	1.58 (1.27–1.97)	9473	9900	95.7	0.68 (0.61–0.75)	1.31 (1.14–1.50)
	Mixed	9201	9338	98.5	0.58 (0.49–0.70)	0.76 (0.64–0.92)	8920	9338	95.5	0.65 (0.59–0.72)	0.78 (0.71–0.87)
	Black	1898	1970	96.3	0.23 (0.18–0.29)	0.99 (0.75–1.33)	1781	1970	**90.4**	0.29 (0.25–0.33)	0.92 (0.77–1.11)
Comorbidity score	0	328,434	331,538	99.1	Baseline	Baseline	32,1268	331,538	96.9	Baseline	Baseline
	1	79,664	80,288	99.2	1.21 (1.11–1.32)	1.17 (1.07–1.28)	77,985	80,288	97.1	1.08 (1.03–1.13)	1.10 (1.05–1.16)
	2	5353	5404	99.1	0.99 (0.76–1.33)	1.03 (0.78–1.39)	5209	5404	96.4	0.85 (0.74–0.99)	0.95 (0.82–1.10)
	3+	2135	2175	98.2	0.50 (0.37–0.70)	0.58 (0.42–0.81)	2078	2175	95.5	0.68 (0.56–0.85)	0.87 (0.71–1.09)
Age first registered with primary care GP in Wales	At birth	358,121	360,620	99.3	Baseline	Baseline	351,458	360,620	97.5	Baseline	Baseline
	Young child (2–3)	33,881	34,335	98.7	0.52 (0.47–0.58)	0.70 (0.63–0.78)	32,798	34,335	95.5	0.56 (0.53–0.59)	0.69 (0.65–0.73)
	Primary school (4–11)	11,535	12,120	95.2	0.14 (0.13–0.15)	0.31 (0.27–0.35)	10,663	12,120	**88.0**	0.19 (0.18–0.20)	0.36 (0.33–0.39)
	Secondary school (12–16)	1609	1761	**91.4**	0.07 (0.06–0.09)	0.16 (0.13–0.20)	1472	1761	**83.6**	0.13 (0.12–0.15)	0.25 (0.22–0.29)
	College (17–18)	672	691	97.3	0.25 (0.16–0.40)	0.37 (0.24–0.61)	645	691	**93.3**	0.37 (0.27–0.50)	0.53 (0.39–0.73)
	University (19–21)	5266	5330	98.8	0.57 (0.45–0.74)	0.70 (0.54–0.91)	5133	5330	96.3	0.68 (0.59–0.79)	0.78 (0.68–0.91)
	Young adult (22–25)	4502	4548	99.0	0.68 (0.52–0.93)	0.84 (0.63–1.15)	4371	4548	96.1	0.64 (0.55–0.75)	0.77 (0.66–0.90)
Mother’s age at delivery	Under 17	2987	3002	99.5	1.69 (1.05–2.95)	1.91 (1.18–3.37)	2905	3002	96.8	0.90 (0.74–1.11)	0.97 (0.79–1.20)
	17–18	14,508	14,610	99.3	1.21 (0.99–1.49)	1.30 (1.05–1.62)	14,143	14,610	96.8	0.91 (0.83–1.01)	0.92 (0.83–1.02)
	19–20	26,875	27,051	99.3	1.30 (1.11–1.53)	1.33 (1.13–1.58)	26,293	27,051	97.2	1.04 (0.97–1.13)	1.03 (0.95–1.13)
	21–25	95,435	96,284	99.1	0.95 (0.87–1.05)	1.02 (0.93–1.12)	93,125	96,284	96.7	0.89 (0.85–0.93)	0.92 (0.88–0.97)
	26–30	125,482	126,548	99.2	Baseline	Baseline	122,849	126,548	97.1	Baseline	Baseline
	31–35	102,444	103,414	99.1	0.90 (0.82–0.98)	0.88 (0.80–0.96)	100,419	103,414	97.1	1.01 (0.96–1.06)	0.99 (0.94–1.04)
	36–40	41,039	41,555	98.8	0.68 (0.61–0.75)	0.70 (0.63–0.78)	40,176	41,555	96.7	0.88 (0.82–0.93)	0.91 (0.85–0.97)
	Over 40	6816	6941	98.2	0.46 (0.39–0.56)	0.60 (0.49–0.73)	6630	6941	95.5	0.64 (0.57–0.72)	0.79 (0.70–0.90)
Birth order	First born	160,101	160,931	99.5	Baseline	Baseline	157,847	160,931	98.1	Baseline	Baseline
	Second born	182,014	183,880	99.0	0.51 (0.47–0.55)	0.74 (0.68–0.81)	177,856	183,880	96.7	0.58 (0.55–0.60)	0.70 (0.66–0.73)
	Third born	48,304	48,845	98.9	0.46 (0.42–0.52)	0.54 (0.49–0.61)	46,895	48,845	96.0	0.47 (0.44–0.50)	0.50 (0.47–0.53)
	Forth born	16,425	16,726	98.2	0.28 (0.25–0.32)	0.37 (0.32–0.43)	15,713	16,726	**93.9**	0.30 (0.28–0.33)	0.35 (0.33–0.38)
	Fifth born	5506	5658	97.3	0.19 (0.16–0.22)	0.27 (0.23–0.33)	5209	5658	**92.1**	0.23 (0.20–0.25)	0.29 (0.26–0.32)
	Sixth or more	3236	3365	96.2	0.13 (0.11–0.16)	0.21 (0.17–0.26)	3020	3365	**89.7**	0.17 (0.15–0.19)	0.23 (0.20–0.26)
Total primary care GP visits 1 September 2020 to 31 August 2021	None	38,114	38,843	98.1	Baseline	Baseline	36,931	38,843	95.1	Baseline	Baseline
	1–2	114,209	115,168	99.2	2.28 (2.07–2.51)	2.18 (1.97–2.41)	111,863	115,168	97.1	1.75 (1.65–1.86)	1.72 (1.62–1.83)
	3–4	72,737	73,290	99.2	2.52 (2.25–2.81)	2.53 (2.25–2.84)	71,298	73,290	97.3	1.85 (1.74–1.98)	1.92 (1.80–2.05)
	5–9	90,044	90,745	99.2	2.46 (2.21–2.73)	2.52 (2.26–2.81)	88,290	90,745	97.3	1.86 (1.75–1.98)	1.98 (1.86–2.11)
	10–14	40,422	40,743	99.2	2.41 (2.11–2.75)	2.64 (2.30–3.04)	39,565	40,743	97.1	1.74 (1.62–1.87)	1.97 (1.82–2.13)
	15–19	23,819	23,992	99.3	2.63 (2.24–3.12)	3.06 (2.57–3.65)	23,298	23,992	97.1	1.74 (1.59–1.90)	2.09 (1.91–2.30)
	20–24	13,989	14,122	99.1	2.01 (1.68–2.43)	2.43 (2.01–2.97)	13,632	14,122	96.5	1.44 (1.30–1.60)	1.82 (1.64–2.03)
	25–49	19,737	19,952	98.9	1.76 (1.51–2.05)	2.31 (1.96–2.74)	19,222	19,952	96.3	1.36 (1.25–1.49)	1.88 (1.71–2.07)
	50+	2515	2550	98.6	1.37 (0.99–1.97)	2.12 (1.51–3.09)	2441	2550	95.7	1.16 (0.96–1.42)	1.90 (1.55–2.35)
Recorded language	No	5525	5863	**94.2**	Baseline	Baseline	5170	5863	**88.2**	Baseline	Baseline
English or Welsh	Yes	410,061	413,542	99.2	7.21 (6.41–8.07)	1.71 (1.44–2.03)	401,370	413,542	97.1	4.42 (4.07–4.79)	1.33 (1.18–1.49)
Ever eligible for	No	296,942	299,262	99.2	Baseline	Baseline	291,750	299,262	97.5	Baseline	Baseline
free school meals	Yes	118,644	120,143	98.8	0.62 (0.58–0.66)	0.73 (0.68–0.79)	114,790	120,143	95.5	0.55 (0.53–0.57)	0.73 (0.70–0.76)
Ever attended a	No	410,837	414,573	99.1	Baseline	Baseline	402,105	414,573	97.0	Baseline	Baseline
special school	Yes	4749	4832	98.3	0.52 (0.42–0.65)	0.67 (0.53–0.85)	4435	4832	91.8	0.35 (0.31–0.38)	0.43 (0.39–0.48)
	None	59,223	60,034	98.6	Baseline	Baseline	57,107	60,034	95.1	Baseline	Baseline
Mother’s highest qualification	A–levels	63,435	63,907	99.3	1.84 (1.64–2.06)	1.12 (0.99–1.27)	62,375	63,907	97.6	2.09 (1.96–2.22)	1.25 (1.17–1.34)
	GCSE/O–Level high grades	85,168	85,779	99.3	1.91 (1.72–2.12)	1.25 (1.12–1.40)	83,527	85,779	97.4	1.90 (1.80–2.01)	1.25 (1.17–1.32)
	GCSE/O–Level any grades	74,792	75,459	99.1	1.54 (1.39–1.70)	1.10 (0.99–1.22)	72,997	75,459	96.7	1.52 (1.44–1.61)	1.11 (1.04–1.17)
	Degree	120,598	121,585	99.2	1.67 (1.52–1.84)	1.10 (0.98–1.23)	118,564	121,585	97.5	2.01 (1.91–2.12)	1.18 (1.11–1.26)
	Apprenticeship	2965	2999	98.9	1.19 (0.86–1.72)	0.83 (0.59–1.20)	2910	2999	97.0	1.68 (1.36–2.09)	1.12 (0.91–1.40)
	Other	9405	9642	97.5	0.54 (0.47–0.63)	0.97 (0.82–1.16)	9060	9642	**94.0**	0.80 (0.73–0.88)	1.06 (0.95–1.17)
Recorded religion	No religion	187,177	188,816	99.1	Baseline	Baseline	18,2806	188,816	96.8	Baseline	Baseline
	Christianity	214,191	216,089	99.1	0.99 (0.92–1.06)	1.11 (1.03–1.19)	209,973	216,089	97.2	1.13 (1.09–1.17)	1.09 (1.05–1.13)
	Buddhism	1093	1146	95.4	0.18 (0.14–0.24)	0.40 (0.29–0.56)	1044	1146	**91.1**	0.34 (0.28–0.42)	0.56 (0.45–0.71)
	Islam	9219	9342	98.7	0.66 (0.55–0.79)	1.45 (1.13–1.87)	8961	9342	95.9	0.77 (0.70–0.86)	1.42 (1.23–1.66)
	Other religions	2344	2412	97.2	0.30 (0.24–0.39)	0.38 (0.30–0.50)	2251	2412	**93.3**	0.46 (0.39–0.54)	0.51 (0.43–0.60)
	Paganism	*	*	*	0.22 (0.15–0.32)	0.32 (0.22–0.48)	729	797	**91.5**	0.35 (0.28–0.46)	0.43 (0.33–0.56)
	Hinduism	*	*	*	1.00 (0.51–2.33)	1.70 (0.83–4.08)	776	803	96.6	0.94 (0.66–1.42)	1.34 (0.90–2.07)
UK Born	Yes	410,890	414,182	99.2	Baseline	Baseline	40,2474	414,182	97.2	Baseline	Baseline
	No	4696	5223	**89.9**	0.07 (0.06–0.08)	0.21 (0.18–0.25)	4066	5223	**77.8**	0.10 (0.10–0.11)	0.23 (0.21–0.26)

* Data suppressed to comply with statistical disclosure policy; Groups with uptake under 95% are indicated with bold text.

## Data Availability

The data used in this study is available from the SAIL Databank at Swansea University, Swansea, UK, which is part of the national e-health records research infrastructure for Wales. All proposals to use SAIL data are subject to review by an independent Information Governance Review Panel (IGRP). Before any data can be accessed, approval must be given by the IGRP. The IGRP gives careful consideration to each project to ensure proper and appropriate use of SAIL data. When access has been approved, it is gained through a privacy-protecting safe haven and remote access system referred to as the SAIL Gateway. SAIL has established an application process to be followed by anyone who would like to access data via SAIL at: https://saildatabank.com/data/apply-to-work-with-the-data/ (accessed on 16 March 2023).

## Supplementary Materials

The following supporting information can be downloaded at: https://www.mdpi.com/article/10.3390/vaccines11030680/s1, Table S1: Uptake of one or two doses of measles-containing vaccine in those aged 4 to 25 years alive and resident in Wales as of 31 August 2021, without a vaccine refusal code, by individual characteristics. Univariable Odds Ratios and 95% Confidence Intervals are also presented. Groups with uptake under 95% are indicated with bold text. Analysis is presented for the whole study cohort.
