# Supplementary material for: Determinants of Equity in Coverage of Measles-Containing Vaccines in Wales, UK, during the Elimination Era
Source: Vaccines (Basel). 2023 Mar 17;11(3):680. doi: 10.3390/vaccines11030680 (PMC10057771; doi:10.3390/vaccines11030680)
Supplement: Supplementary file 1 [file vaccines-11-00680-s001.zip › vaccines-2246140-supplementary.pdf]

Supplementary Table S1. Uptake of one or two doses of measles-containing vaccine in those aged 4 to 25 years alive and resident in Wales as at 31 August 2021, without a vaccine refusal code, by individual characteristics. Univariable Odds Ratios and 95% Confidence Intervals are also presented. Groups with uptake under 95% are indicated with bold text. Analysis is presented for the whole study cohort.

Characteristic	Category	One dose of measles-containing vaccine (2 to 25-year-olds)				Two doses of measles-containing vaccine (4 to 25-year-olds)			
		Vaccinated	Population	Uptake	Odds Ratio (95% CI)	Vaccinated	Population	Uptake	Odds Ratio (95% CI)
Gender	Male	322953	331333	97.5	Baseline	288712	306527	**94.2**	Baseline
	Female	305572	312874	97.7	1.09 (1.05-1.12)	273985	289294	**94.7**	1.10 (1.08-1.13)
Age cohort	Young child (2-3)	46734	48386	96.6	0.64 (0.61 - 0.68)	N/a	N/a		
	Primary school (4-11)	220869	225882	97.8	Baseline	213543	225882	**94.5**	Baseline
	Secondary school (12-16)	141487	144962	97.6	0.92 (0.88 - 0.97)	137865	144962	95.1	1.22 (1.09 - 1.16)
	College (17-18)	51925	53411	97.2	0.79 (0.75 - 0.84)	50353	53411	**94.3**	0.95 (0.91 - 0.99)
	University (19-21)	69204	71067	97.4	0.84 (0.80 - 0.89)	66689	71067	**93.8**	0.88 (0.85 - 0.91)
	Young adults (22-25)	98306	100499	97.8	1.02 (0.97 - 1.07)	94247	100499	**93.8**	0.87 (0.84 - 0.90)
Health board of residence	HB1	126605	129008	98.1	Baseline	114841	119559	96.1	Baseline
	HB2	86092	88675	97.1	0.63 (0.60-0.67)	76489	81930	**93.4**	0.58 (0.55-0.60)
	HB3	66005	68067	97.0	0.61 (0.57-0.64)	59104	63174	**93.6**	0.60 (0.57-0.62)
	HB4	10867	11157	97.4	0.71 (0.63-0.81)	9776	10365	**94.3**	0.68 (0.62-0.75)
	HB5	112457	114820	97.9	0.90 (0.85-0.96)	100300	106022	**94.6**	0.72 (0.69-0.75)
	HB6	118608	121427	97.7	0.80 (0.76-0.84)	106272	112274	**94.7**	0.73 (0.70-0.76)
	HB7	107891	111053	97.2	0.65 (0.61-0.68)	95915	102497	**93.6**	0.60 (0.58-0.62)
Urban/rural residence	Rural	167637	170966	98.1	Baseline	151717	158769	95.6	Baseline
	Urban	460888	473241	97.4	0.74 (0.71-0.77)	410980	437052	**94.0**	0.73 (0.71-0.75)
Deprivation quintile of residence	Most deprived	156244	161365	96.8	Baseline	137637	148583	**92.6**	Baseline
	2	132238	135455	97.6	1.35 (1.29-1.41)	118200	125111	**94.5**	1.36 (1.32-1.40)
	3	114339	117179	97.6	1.32 (1.26-1.38)	102476	108317	**94.6**	1.40 (1.35-1.44)
	4	107384	109686	97.9	1.53 (1.45-1.61)	96969	101701	95.3	1.63 (1.57-1.69)
	Least deprived	118320	120522	98.2	1.76 (1.67-1.85)	107415	112109	95.8	1.82 (1.76-1.89)

Premature birth	No	532270	539189	98.7	Baseline	478081	496521	96.3	Baseline
	Yes	42682	43235	98.7	1.00 (0.92-1.10)	37967	39558	96.0	0.92 (0.87-0.97)
	Unknown	53573	61783	**86.7**	-	46649	59742	**78.1**	-
Maternal smoker	No	151121	152720	99.0	Baseline	135418	139150	97.3	Baseline
	Yes	40463	41027	98.6	0.76 (0.69-0.84)	36409	38115	95.5	0.59 (0.55-0.62)
	Unknown	436941	450460	97.0	-	390870	418556	**93.4**	-
Ethnic group	White	512072	520137	98.4	Baseline	494533	516375	95.8	Baseline
	Other	5338	6283	**85.0**	0.09 (0.08-0.10)	4575	6061	**75.5**	0.14 (0.13-0.14)
	Asian	16381	17133	95.6	0.34 (0.32-0.37)	15348	16820	**91.2**	0.46 (0.44-0.49)
	Mixed	15528	16169	96.0	0.38 (0.35-0.41)	14606	15953	**91.6**	0.48 (0.45-0.51)
	Unknown	75026	79870	**93.9**	-	29911	36089	**82.9**	-
	Black	4180	4615	**90.6**	0.15 (0.14-0.17)	3724	4523	**82.3**	0.21 (0.19-0.22)
Learning disability	No	625181	640778	97.6	Baseline	559620	592455	**94.5**	Baseline
	Yes	3344	3429	97.5	0.98 (0.80-1.23)	3077	3366	**91.4**	0.62 (0.55-0.71)
Sight-loss	No	626900	642556	97.6	Baseline	561160	594208	**94.4**	Baseline
	Yes	1625	1651	98.4	1.56 (1.08-2.36)	1537	1613	95.3	1.19 (0.95-1.51)
Hearing-loss	No	587209	602418	97.5	Baseline	522834	554578	**94.3**	Baseline
	Yes	41316	41789	98.9	2.26 (2.07-2.48)	39863	41243	96.7	1.75 (1.66-1.85)
Co-morbidity score	0	512258	526379	97.3	Baseline	451772	480263	**94.1**	Baseline
	1	106178	107558	98.7	2.12 (2.01-2.24)	101423	105545	96.1	1.55 (1.50-1.60)
	2	7302	7425	98.3	1.64 (1.38-1.97)	6857	7215	95.0	1.21 (1.09-1.35)
	3+	2787	2845	98.0	1.32 (1.03-1.74)	2645	2798	**94.5**	1.09 (0.93-1.29)
Age first registered with primary care GP in Wales	At birth	511628	517554	98.9	Baseline	457432	473459	96.6	Baseline
	Young child (1-3)	59968	61637	97.3	0.42 (0.39 - 0.44)	53622	57346	**93.5**	0.50 (0.49 - 0.52)
	Primary school (4-11)	34937	39861	**87.6**	0.08 (0.08 - 0.09)	31401	39861	**78.8**	0.13 (0.13 - 0.13)
	Secondary school (12-16)	7760	10172	**76.3**	0.04 (0.04 - 0.04)	6714	10172	**66.0**	0.07 (0.07 - 0.07)
	College (17-18)	1712	2112	**81.1**	0.05 (0.04 - 0.06)	1539	2112	**72.9**	0.09 (0.09 - 0.10)
	University (19-21)	6903	7130	96.8	0.35 (0.31 - 0.40)	6598	7130	**92.5**	0.43 (0.40 - 0.48)

	Young adult (22-25)	5617	5741	97.8	0.52 (0.44 - 0.63)	5391	5741	**93.9**	0.54 (0.48 - 0.60)
Mothers age at delivery	Under 17	5122	5241	97.7	0.82 (0.68-0.99)	4815	5114	**94.2**	0.79 (0.70-0.89)
	17-18	22763	23184	98.2	1.03 (0.93-1.14)	21227	22353	95.0	0.92 (0.86-0.98)
	19-20	40786	41503	98.3	1.08 (1.00-1.17)	37654	39425	95.5	1.04 (0.99-1.10)
	21-25	140031	142749	98.1	0.98 (0.93-1.03)	126325	133093	**94.9**	0.91 (0.88-0.94)
	26-30	179326	182735	98.1	Baseline	159845	167660	95.3	Baseline
	31-35	145484	148332	98.1	0.97 (0.92-1.02)	129221	135264	95.5	1.05 (1.01-1.08)
	36-40	60235	61641	97.7	0.81 (0.77-0.87)	53192	55981	95.0	0.93 (0.89-0.97)
	Over 40	34778	38822	**89.6**	0.16 (0.16-0.17)	30418	36931	**82.4**	0.23 (0.22-0.24)
Birth order	First born	227676	229767	99.1	Baseline	207864	213812	97.2	Baseline
	Second born	168229	170200	98.8	0.78 (0.74-0.83)	150965	156332	96.6	0.80 (0.78-0.84)
	Third born	68252	69437	98.3	0.53 (0.49-0.57)	60870	64162	**94.9**	0.53 (0.51-0.55)
	Forth born	24167	24831	97.3	0.33 (0.31-0.37)	21154	22873	**92.5**	0.35 (0.33-0.37)
	Fifth born	8518	8851	96.2	0.23 (0.21-0.26)	7357	8132	**90.5**	0.27 (0.25-0.29)
	Sixth or more	5487	5804	**94.5**	0.16 (0.14-0.18)	4640	5270	**88.0**	0.21 (0.19-0.23)
	Unknown	126196	135317	**93.3**	-	109847	125240	**87.7**	-
Recorded language English or Welsh	No	8173	9147	**89.4**	Baseline	7271	8961	**81.1**	Baseline
	Yes	582599	590815	98.6	8.45 (7.88-9.06)	523439	544991	96.0	5.65 (5.34-5.96)
	Unknown	37753	44245	**85.3**	-	31987	41869	**76.4**	-
Total primary care GP visits 01/09/2020 to 31/08/2021	None	59987	63848	94.0	Baseline	53951	61281	**88.0**	Baseline
	1-2	164529	168730	97.5	2.52 (2.41-2.64)	152549	160991	**94.8**	2.46 (2.38-2.54)
	3-4	111531	114098	97.8	2.80 (2.66-2.94)	99485	104731	95.0	2.58 (2.48-2.67)
	5-9	144593	147332	98.1	3.40 (3.23-3.57)	124742	130716	95.4	2.84 (2.74-2.94)
	10-14	62750	63755	98.4	4.02 (3.75-4.31)	54474	56957	95.6	2.98 (2.84-3.12)
	15-19	34843	35345	98.6	4.47 (4.07-4.91)	31167	32544	95.8	3.08 (2.90-3.26)
	20-24	19818	20119	98.5	4.24 (3.77-4.78)	18062	18897	95.6	2.94 (2.73-3.17)
	25-49	27023	27458	98.4	4.00 (3.62-4.43)	25036	26294	95.2	2.70 (2.54-2.88)
	50+	3451	3522	98.0	3.13 (2.49-4.00)	3231	3410	**94.8**	2.45 (2.11-2.87)

Ever eligible for free school meals	No	375348	381941	98.3	Baseline	366499	381941	96.0	Baseline
	Yes	168934	172397	98.0	0.86 (0.82-0.89)	162129	172397	**94.0**	0.67 (0.65-0.68)
	Too young	46734	48386	96.6	-	N/a	N/a	N/a	-
	Unknown	37509	41483	**90.4**	-	34069	41483	**82.1**	-
Ever attended a special school	No	537716	547626	98.2	Baseline	522515	547626	95.4	Baseline
	Yes	6566	6712	97.8	0.83 (0.71-0.98)	6113	6712	**91.1**	0.49 (0.45-0.53)
	Too young	46734	48386	96.6	-	N/a	N/a	N/a	-
	Unknown	37509	41483	**90.4**	-	34069	41483	**82.1**	-
Ever excluded from school	No	514916	524387	98.2	Baseline	500456	524387	95.4	Baseline
	Yes	29365	29950	98.0	0.92 (0.85-1.01)	28171	29950	**94.1**	0.76 (0.72-0.80)
	Too young	46734	48386	96.6	-	N/a	N/a	N/a	-
	Unknown	37510	41484	**90.4**	-	34070	41484	**82.1**	-
UK Born	Yes	585022	592771	98.7	Baseline	525993	546671	96.2	Baseline
	No	8044	9611	**83.7**	0.07 (0.06-0.07)	6679	9434	**70.8**	0.10 (0.09-0.10)
	Unknown	35459	41825	**84.8**	-	30025	39716	**75.6**	-
Mothers highest qualification	None	67273	68404	98.3	Baseline	62218	65702	**94.7**	Baseline
	A-levels	74917	75607	99.1	1.83 (1.66-2.01)	68504	70398	97.3	2.03 (1.91-2.14)
	GCSE/O-Level high grades	99175	100106	99.1	1.79 (1.64-1.95)	91236	93988	97.1	1.86 (1.76-1.95)
	GCSE/O-Level any grades	86051	87030	98.9	1.48 (1.36-1.61)	79594	82565	96.4	1.50 (1.43-1.58)
	Unknown	144762	154892	**93.5**	-	116405	133744	**87.0**	-
	Degree	141845	143273	99.0	1.67 (1.54-1.81)	131434	135228	97.2	1.94 (1.85-2.03)
	Apprenticeship	3601	3647	98.7	1.32 (0.99-1.80)	3254	3367	96.6	1.61 (1.34-1.96)
	Other	10901	11248	96.9	0.53 (0.47-0.60)	10052	10829	**92.8**	0.72 (0.67-0.79)
Recorded religion	No religion	223619	226128	98.9	Baseline	203422	210887	96.5	Baseline
	Christianity	253174	255982	98.9	1.01 (0.96-1.07)	232740	240517	96.8	1.10 (1.06-1.13)
	Buddhism	1320	1396	**94.6**	0.19 (0.16-0.25)	1193	1340	**89.0**	0.30 (0.25-0.36)
	Unknown	134301	144207	**93.1**	-	110499	127322	**86.8**	-

	Islam	11280	11520	97.9	0.53 (0.46-0.60)	10441	11019	**94.8**	0.66 (0.61-0.72)
	Other religions	2842	2933	96.9	0.35 (0.29-0.44)	2575	2781	**92.6**	0.46 (0.40-0.53)
	Paganism	869	910	95.5	0.24 (0.18-0.33)	779	860	**90.6**	0.35 (0.28-0.45)
	Hinduism	1120	1131	99.0	1.14 (0.66-2.21)	1048	1095	95.7	0.82 (0.62-1.11)
Had pneumococcal vaccine	No	31227	40106	**77.9**	Baseline	26668	37696	**70.7**	Baseline
	Yes	350740	351302	99.8	177.45 (162.99-193.60)	298337	305326	97.7	17.65 (17.09-18.24)
	Born before introduction	246558	252799	97.5	-	237692	252799	**94.0**	-
Had three doses of pertussis vaccine	No	14561	25600	**56.9**	Baseline	9566	23385	**40.9**	Baseline
	Yes	613964	618607	99.2	100.25 (96.52-104.14)	553131	572436	96.6	41.39 (40.18-42.64)
Had two doses of rotavirus vaccine	No	9277	11960	**77.6**	Baseline	5973	8584	**69.6**	Baseline
	Yes	142752	144248	99.0	27.60 (25.82-29.50)	95261	99238	96.0	10.47 (9.90-11.07)
	Born before introduction	476496	487999	97.6	-	461463	487999	**94.6**	-
